# Resolving the “Thick-Wall Challenge” in *Haematococcus pluvialis*: From Metabolic Reprogramming to Clinical Translation

**DOI:** 10.3390/microorganisms14010253

**Published:** 2026-01-21

**Authors:** Tao Chen, Xun Zhu, Qiang Liao

**Affiliations:** 1 Key Laboratory of Low-Grade Energy Utilization Technologies and Systems, Chongqing University, Ministry of Education, Chongqing 400044, China; 2 Institute of Engineering Thermophysics, School of Energy and Power Engineering, Chongqing University, Chongqing 400044, China

**Keywords:** *Haematococcus pluvialis*, astaxanthin, thick-wall challenge, biological activity, biorefinery, microfluidics, solvent extraction, metabolic regulation

## Abstract

Astaxanthin, derived from *Haematococcus pluvialis*, is a potent antioxidant with significant therapeutic potential. However, its large-scale commercialization is hindered by the “thick-wall challenge”, a phenomenon where the stress conditions required for astaxanthin accumulation also trigger the formation of resistant secondary cell walls. This challenge complicates extraction and reduces bioaccessibility, thereby increasing production costs. Recent advancements have focused on uncoupling astaxanthin biosynthesis from cell wall reinforcement, utilizing metabolic engineering and strain selection to reduce wall formation while maintaining high yields. Furthermore, green extraction techniques, such as electrotechnologies and ionic liquids, are being explored to improve efficiency and environmental sustainability. This review synthesizes these innovations, including biorefinery systems that maximize biomass valorization, and discusses emerging clinical applications. We highlight the challenges in bridging the gap between laboratory successes and clinical translation, and suggest future directions for resolving the thick-wall challenge, advancing astaxanthin production, and expanding its therapeutic uses in nutraceuticals and pharmaceuticals.

## 1. Introduction

In current life science and medical research, oxidative stress is widely acknowledged as a core health challenge throughout the human lifespan. It describes a pathological state where the body’s endogenous antioxidant defense systems are overwhelmed by the production of reactive oxygen species (ROS) and reactive nitrogen species (RNS), disrupting redox homeostasis [1,2]. This imbalance triggers a cascade of molecular and cellular damage: lipid peroxidation impairs cell membrane integrity and fluidity; oxidative modification of proteins undermines their enzymatic activity and structural stability; and oxidative DNA damage promotes gene mutations and malignant cellular transformation [3,4]. A growing body of epidemiological and clinical studies have shown that chronic oxidative stress serves as a common pathological foundation for numerous diseases, including cardiovascular disorders (such as atherosclerosis), neurodegenerative diseases (such as Alzheimer’s and Parkinson’s diseases), metabolic syndromes (such as diabetes and obesity), and various cancers [5,6,7]. As the global population ages and the burden of exogenous oxidative stress intensifies—driven by environmental pollution, radiation exposure, and unhealthy dietary habits—there is an urgent need for highly effective and safe antioxidants suitable for long-term use in preventive medicine and nutritional interventions [8,9]. Against this backdrop, carotenoids have become a key focus of antioxidant research, thanks to their unique conjugated polyene structures that allow them to efficiently quench singlet oxygen and scavenge free radicals. Among the over 700 identified carotenoids, astaxanthin stands out for its exceptional antioxidant capacity, earning it widespread recognition as a “super antioxidant” or the “king of carotenoids” [10].

**Figure 1:**
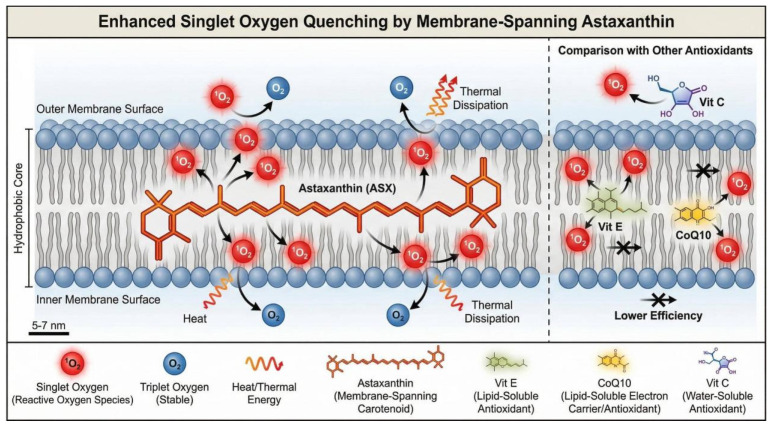
Transmembrane Integration and Quenching Kinetics: Schematic illustrating the structure of astaxanthin (ASX) molecules integrated into a phospholipid bilayer. Due to its amphiphilic structure, ASX spans the entire hydrophobic core region, with its polar terminal groups binding to the hydrophilic surfaces of the inner and outer leaflets, while the hydrophobic polyene backbone penetrates the lipid core. This perpendicular orientation forms a “molecular bridge” that stabilizes the bilayer structure. The illustration depicts the process by which ASX’s conjugated polyene chain scavenges high-energy singlet oxygen: after absorbing excitation energy, it undergoes physical quenching, releasing thermal energy (heat) while reducing the oxygen back to its ground-state triplet state, leaving the ASX molecule undamaged. Antioxidant efficacy comparison: Spatial distribution of ASX versus conventional antioxidants. Vitamin C is confined to the aqueous phase (hydrophilic) and cannot interact with lipid peroxy radicals in the core region. Vitamin E and coenzyme Q10 (CoQ10), though hydrophobic, exhibit disordered lateral distribution within the bilayer. In contrast, ASX’s rigid transmembrane arrangement creates a superior cross-sectional contact area for intercepting reactive oxygen species, providing stronger antioxidant stress protection compared to non-transmembrane antioxidants.

Astaxanthin (3,3′-dihydroxy-4,4′-diketo-β,β′-carotene) is a member of the xanthophyll subgroup of carotenoids. Its molecular structure consists of a central polyene chain with an extended system of conjugated double bonds, flanked by two β-ionone rings. Unlike structurally similar carotenoids, such as β-carotene and lutein, astaxanthin is unique in having both hydroxyl (–OH) and keto (=O) functional groups on each terminal ring—a feature that gives it unique physicochemical properties [11]. The polar functional groups are the basis for astaxanthin’s remarkable biophysical behaviors, particularly its ability to span biological membranes. Within the phospholipid bilayer, astaxanthin adopts a transmembrane arrangement: its polar end groups is accommodated by the hydrophilic surfaces of the inner and outer membrane leaflets, while the hydrophobic polyene backbone penetrates the lipid core. This structure not only enhances membrane stability but also allows astaxanthin to neutralize free radicals on both sides of the membrane, thereby effectively halting lipid peroxidation chain reactions [12]. Its singlet oxygen quenching capacity, measured via chemiluminescence and ESR spin-trapping assays, is reported to be over 550 times that of vitamin E, 800 times that of coenzyme Q10, more than 10 times that of β-carotene, and up to 6000 times that of vitamin C [13]. These ratio values reflect relative activity under standardized in vitro conditions, and in vivo efficacy may vary due to bioaccessibility differences. This exceptional antioxidant activity supports its broad therapeutic potential, including protection against UV-induced skin photoageing [14,15], retinal photodamage, and exercise-related oxidative stress in skeletal muscle [16] (Figure 1).

Despite the fast-growing demand for astaxanthin, commercially available products are not biologically equivalent. Currently, commercial astaxanthin is mainly derived from three sources: chemical synthesis, fermentation by Phaffia rhodozyma, and cultivation of *Haematococcus pluvialis*. Chemically synthesized astaxanthin dominates the animal feed market—especially in aquaculture, such as for salmon pigmentation—thanks to its low production cost and short manufacturing cycle. However, synthetic astaxanthin is produced as a racemic mixture, typically consisting of (3S,3′S), meso (3R,3′S), and (3R,3′R) stereoisomers in an approximate 1:2:1 ratio, and exists mostly in the free, non-esterified form. Furthermore, concerns remain about residual petrochemical precursors and the long-term safety of synthetic astaxanthin for human consumption, limiting its application in nutrition and pharmaceuticals [17,18]. In contrast, natural astaxanthin from *Haematococcus pluvialis* is widely recognized as the highest-quality source. Stereochemically, it exists almost exclusively as the all-trans (3S,3′S) isomer, which has superior biological activity and is more efficiently absorbed and metabolized in the body. Additionally, natural astaxanthin is predominantly present as monoesters and diesters, where the astaxanthin backbone is esterified with fatty acids. Mounting evidence shows that esterified astaxanthin has enhanced thermal stability and improved emulsifying properties, which facilitate its incorporation into chylomicrons and result in higher bioaccessibility compared to the free form [19,20,21]. For this reason, the development of high-end nutraceuticals, cosmetics, and pharmaceutical products for human health has focused heavily on natural astaxanthin sourced from *Haematococcus pluvialis*.

*Haematococcus pluvialis* is a unicellular freshwater green alga, well-known for its remarkable ability to synthesize and accumulate astaxanthin—with concentrations reaching 4–5% of its dry cell weight [22]. This impressive accumulation capacity stems from its unique survival adaptation: when exposed to stress conditions such as intense light, nutrient deprivation (e.g., nitrogen or phosphorus deficiency), or high salt environments, the alga undergoes profound morphological and metabolic shifts. The cells transform from motile, green vegetative forms into larger, non-motile cells that develop thick-walled sporangia and produce substantial amounts of red astaxanthin. This process acts as a protective mechanism: astaxanthin functions both as a “light shield” and an antioxidant, safeguarding the organism’s genetic material [23,24,25].

However, this biological survival advantage creates a major technical hurdle for industrial application. To endure stressful environments, *Haematococcus pluvialis* not only accumulates astaxanthin but also synthesizes a thick secondary cell wall—around 2 μm in thickness—that is rigid and resistant to both acid and alkali. This cell wall is primarily composed of cellulose and the biopolymer sporopollenin [26,27].

This gives rise to the “*Haematococcus pluvialis* challenge”: achieving high astaxanthin yields requires imposing strong stress, which in turn triggers extensive cell wall thickening. For practical applications, this thick wall severely reduces the digestibility and bioaccessibility of algal powder when consumed directly. More critically, it complicates the downstream extraction of astaxanthin. Traditional mechanical disruption methods, such as high-pressure homogenization, demand substantial energy input; worse, the frictional heat generated during these processes can degrade heat-sensitive astaxanthin. Additionally, using organic solvents like acetone or ethyl acetate to boost extraction efficiency raises concerns about environmental pollution and residual solvents in the final product [28,29]. *Haematococcus pluvialis* faces other challenges in large-scale cultivation too. It has a relatively long growth cycle, usually exceeding 20 days. In open-pond cultures, it is also highly susceptible to contamination by faster-growing algae, fungi, or protozoa—a form of “biological predation” that often leads to complete culture collapse [30,31]. The combined effects of unstable cultivation conditions and high extraction costs result in the high market price of natural astaxanthin, limiting its widespread adoption. In summary, while *Haematococcus pluvialis*-derived astaxanthin offers exceptional biological activity, the core challenges lie in resolving this “thick-wall barrier,” developing green and cost-effective production and extraction technologies, and further clarifying its mechanisms of action in complex disease models [32,33,34]. This review will address these critical issues and summarize recent technological innovations and research advances in the field.

## 2. Core Progress: Innovations in Production and Extraction Technologies

The main hurdle to industrializing *Haematococcus pluvialis* is the clash between its natural biological defense mechanisms and the needs of commercial production. The efficient accumulation of astaxanthin usually comes with the formation of tough, recalcitrant cell walls, which increases downstream processing costs and reduces bioaccessibility. Recent research has gone beyond optimizing individual steps, adopting a full value chain approach instead. The focus has shifted to regulating metabolic fluxes and developing eco-friendly separation technologies to change the current paradigm for astaxanthin production and extraction.

*Haematococcus pluvialis* cultivation involves a “two-step method”: a green growth phase to rapidly build up biomass, followed by a red induction phase. During induction, stress conditions like intense light or nitrogen deficiency trigger astaxanthin synthesis. However, these stressors also activate the alga’s cellular defense mechanisms, leading to the thickening of secondary cell walls. Breaking this natural link between “thick walls” and “high yields” has become a key breakthrough point in recent upstream research [35]. A pioneering study by Zhang et al. (2023) developed a taurine-based regulatory strategy that selectively induces astaxanthin synthesis without prompting cell wall thickening [36]. Taurine activates the transcription factor *CrMYB1*, which binds to the promoters of *BKT* (β-carotene ketolase) and *CHY* (β-carotene hydroxylase), This accelerates the conversion of *β-carotene* to astaxanthin. It inhibits the expression of genes encoding cellulose synthase (*CELA*, *CELB*) and sporopollenin synthase (*SPOA*, *SPOB*) by suppressing the *MAPK* signaling pathway. Taurine acts as an osmolyte and antioxidant, scavenging ROS generated under high-light stress. This reduces oxidative damage to carotenoid biosynthesis enzymes and prevents the activation of stress-responsive cell wall thickening pathways. When *Haematococcus pluvialis* culture medium, adding GSH, glutamate, pyroglutamate, GABA, 2-ketoglutarate, sucrose, raffinose, glucose, succinate, flavonoids, uracil, and adenine, to measure changes in astaxanthin yield and cell density following addition. The study revealed that the biosynthetic pathways of the stachyose family oligosaccharides, glutathione metabolism, pyrimidine and purine metabolism, the TCA cycle, and phospholipid metabolism are associated with astaxanthin biosynthesis. It also highlighted the metabolic shift from primary metabolism to astaxanthin biosynthesis [37]. As a result, the cells accumulated high levels of astaxanthin while retaining relatively thin cell walls. These “parenchyma-like cells” eliminate the need for mechanical wall disruption; using conventional solvents (dimethyl sulfoxide or ethanol), astaxanthin recovery rates can reach up to 97%, compared to a mere 3% with traditional methods. Another approach focuses on selecting or breeding algal strains that naturally avoid forming thick-walled sporangia. Li et al. (2017) [38] identified the biomass productivity of *Haematococcus pluvialis* mutants induced by 60Co-γ radiation and acclimated to 15% CO_2_ reached 0.66 g L^−1^ d^−1^. The maximum specific growth rate was 26% higher than that of green cells. The CO_2_ fixation rate of the mutants reached 2.57 g L^−1^ d^−1^, which was 24 times higher than under ambient air conditions and 6 times higher than that of green cells . Using high-pressure cell disruption technology, researchers successfully disrupted cell wall structures, achieving a maximum protein extraction rate of 73% at pH 7. This directly demonstrates the critical importance of cell wall disruption for the release of cellular contents [39]. The CRISPR/Cas9 system has emerged as the most widely applied tool for targeted genetic modification in *H. pluvialis*, enabling both knockout of cell wall-related genes and overexpression of carotenoid biosynthesis genes. Xu et al. (2022) [40] successfully knocked down the *manA* gene, which encodes mannan synthase—a key enzyme in the synthesis of cell wall polysaccharides. The resulting mutant strain exhibited a 60% reduction in cell wall thickness (while maintaining an astaxanthin content of 42.3 mg/g dry weight, comparable to the wild type. Mechanistically, the suppression of *manA* reduced the accumulation of mannan in the secondary cell wall, eliminating the need for energy-intensive mechanical disruption during extraction ).

Traditional plate-based screening methods are inefficient when it comes to rapidly identifying high-yield traits from large algal populations. To address this, Jia et al. (2023) [41] developed a cell-sorting technology based on an ultrastretchable microfluidic chip. This system leverages elasto-inertial forces to continuously sort cells based on subtle differences in size. As *Haematococcus pluvialis* accumulates more astaxanthin, its cell volume increases; the system can dynamically adjust the sorting threshold by stretching the microchannels, enabling non-destructive, high-throughput enrichment of large, high-yield cells . This innovation provides robust hardware support for targeted evolution and genetic improvement of *Haematococcus pluvialis* strains [42,43]. Beyond photoautotrophic cultivation, mixotrophic or heterotrophic growth using organic carbon sources has emerged as an effective strategy to boost biomass production and reduce costs. Sipaúba-Tavares et al. (2022) [44] explored the feasibility of using agricultural waste—specifically sugarcane molasses—as an alternative carbon source. Their findings showed that pretreated (hydrolyzed) molasses, when combined with an NPK inorganic fertilizer medium, significantly promoted *Haematococcus pluvialis* growth and protein accumulation at a much lower cost than the standard BG-11 medium . Additionally, to enhance the bioactivity of the final product, Serwotka-Suszczak et al. (2019) [24] developed a magnesium ion (Mg(II)) bioaccumulation approach. Adding magnesium salts at specific concentrations to the culture medium not only enhanced algal metabolic activity but also increased the efficacy of the resulting algal extract in subsequent experiments targeting insulin resistance .

Table 1 provides an evidence-mapped overview of upstream strategies proposed to mitigate the thick-wall challenge in *H. pluvialis*. In the following sections, we synthesize these strategies by grouping them into cultivation modes/engineering, screening and strain improvement, and metabolic interventions, and we explicitly discuss the proposed mechanisms and practical constraints indicated in the primary studies underlying each table entry.

When upstream strategies cannot fully overcome the recalcitrance of *Haematococcus pluvialis* cell walls, efficient downstream disruption becomes essential. Conventional methods, such as high-pressure homogenization and bead milling, are effective, but they have drawbacks: they are energy-intensive and tend to produce frictional heat, which can degrade the heat-sensitive astaxanthin [50,51]. Therefore, emerging downstream technologies are increasingly adopting milder, more selective, and energy-efficient solutions [52]. Non-thermal electrical technologies, including pulsed electric fields (PEF) and high-voltage discharge, have attracted growing attention in microalgal processing. Gherabli et al. (2023) [22] reviewed their application in *Haematococcus pluvialis* extraction, highlighting that electroporation creates nanoscale pores to facilitate solvent penetration without complete cell rupture, reducing energy consumption and cell debris interference [53,54]. However, critical trade-offs exist: (i) Chemical stability risks: Localized reactive oxygen species (ROS) generated by high-voltage pulses promote lipid peroxidation and cis-isomerization of astaxanthin, especially at pulse intensities exceeding 40 kV/cm; (ii) Scalability limits: Lab-scale throughput (10–100 L/h) is difficult to upscale due to challenges in maintaining uniform electric fields and heat dissipation in large reactors; (iii) Energy balance: While PEF reduces energy use by 30–50% per kg biomass compared to high-pressure homogenization, the energy required for cooling and electrode maintenance offsets partial savings [55]. Optimizing pulse parameters is essential to balance extraction efficiency and chemical integrity. Ionic liquids (ILs) have emerged as promising ‘green solvents’ due to their tunable structures and strong solvating power. Subsequent hexane extraction achieved astaxanthin recovery rates exceeding 99% [56], and room-temperature operation avoids thermal degradation of astaxanthin—a critical advantage for this heat-sensitive compound [57]. However, practical application faces non-negligible constraints: (i) Chemical integrity risks: Anions such as HSO_4_^−^ in imidazolium-based ILs may induce ester hydrolysis or cis-isomerization of all-trans astaxanthin, especially under prolonged mixing; (ii) Toxicological and residual concerns: Trace IL residues (<10 ppm) from long-alkyl-chain imidazolium ILs pose potential hepatotoxicity, requiring energy-intensive purification for food/pharmaceutical use; (iii) Scalability challenges: IL recycling efficiency declines by ~40% after 3–5 cycles due to solvent degradation, and their corrosiveness to stainless steel necessitates inert materials for reactor construction, increasing capital costs. These trade-offs limit ILs to high-value niche applications rather than large-scale commercial production.

Conventional organic solvents like acetone and ethyl acetate deliver high extraction efficiency, but their flammability, toxicity, and environmental risks remain major concerns [58,59]. In recent years, supramolecular solvent systems have driven a paradigm shift toward greener extraction technologies. Deep eutectic solvents (DESs)—typically composed of hydrogen bond acceptors and donors are valued for their low toxicity, biodegradability, and cost-effectiveness. By leveraging the differential partitioning behaviors of target compounds, the system achieved a separation specificity coefficient of 48.31 between astaxanthin monoesters and free astaxanthin. This enabled simultaneous extraction and preliminary purification; importantly, the solvent system remained recyclable for at least three cycles, highlighting its industrial application potential [60].

## 3. Process Innovation: Solvent-Free Extraction and Biorefinery Integration

**Figure 2:**
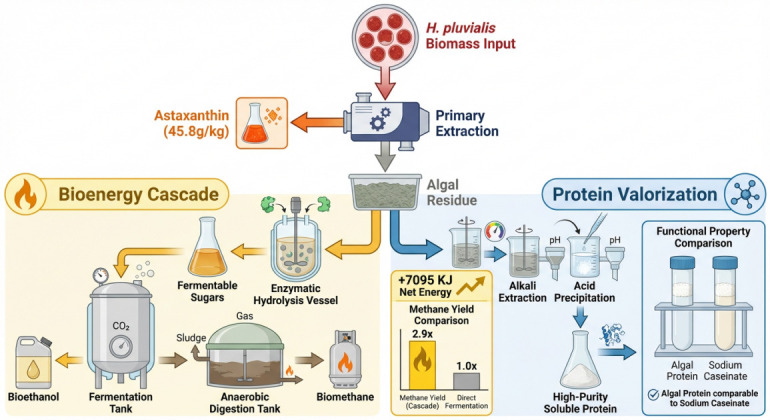
After primary extraction of *Haematococcus pluvialis* algal broth, astaxanthin is selectively recovered as a high-value product (45.8 g/kg biomass, based on sequential solvent-free extraction followed by enzymatic hydrolysis of residues), while the residual algal biomass is directed toward two complementary pathways. For bioenergy, enzymatic hydrolysis converts residual polysaccharides into fermentable sugars, which are then fermented into bio-ethanol. Anaerobic digestion of sludge produces biogas methane. This energy cascade system achieves a net energy gain of approximately +7095 kJ, with methane yield reaching 2.9 times (calculated via anaerobic digestion of lipid-extracted biomass) that of direct fermentation methods. The protein valorization pathway employs alkali extraction and acid precipitation to isolate high-purity algal protein, whose functional properties are validated against commercial protein concentrate and isolate benchmarks. By integrating pigment extraction, renewable bio-energy production, and functional protein recovery, this approach maximizes the economic value and energy recovery of *Haematococcus pluvialis* biomass.

Driven by green chemistry principles and economic sustainability goals, solvent-free extraction strategies and biorefinery concepts are increasingly reshaping the industrial landscape of *H. pluvialis* processing [61]. Patel et al. (2022) [62] and Young Lee et al. (2024) [63] independently proposed an innovative oil-partitioning approach. After cell disruption via homogenization or chemical conditioning, edible vegetable oils are added. Due to its high lipophilicity, astaxanthin spontaneously migrates into the oil phase. Young Lee et al. demonstrated that by optimizing salt concentration and using propylene glycol to assist demulsification, wet algal sludge could be converted into cooking oil containing 1.88% astaxanthin. This method eliminates energy-intensive drying and solvent recovery steps . The resulting astaxanthin-enriched oil is directly suitable for food applications [64,65] (Figure 2).

To avoid overinterpreting headline extraction numbers across heterogeneous studies, we evaluate downstream technologies using a consistent set of criteria: (i) chemical integrity of astaxanthin during processing (oxidation and isomerization risks), (ii) solvent safety and regulatory acceptability for the intended product category, (iii) scalability constraints including throughput, energy demand, and solvent recovery, and (iv) compatibility with the target product form (whole biomass, crude extract/oleoresin, or purified compound; free vs. esterified astaxanthin). We apply these criteria explicitly when discussing each technology summarized in Table 2.

## 4. Core Progress: Biological Activity and Delivery Systems

Advances in molecular mechanism research and extraction-purification technologies have extended academic interest in natural astaxanthin from *Haematococcus pluvialis* beyond its traditional classification as a simple antioxidant [70,71]. Astaxanthin is now characterized as a multi-target, multi-system bioactive compound with diverse pharmacological functions [72]. To overcome its inherent physicochemical limitations, such as poor water solubility and susceptibility to oxidative degradation, the development of advanced delivery systems has become a key research focus [73]. Natural astaxanthin exerts regulatory effects across multiple physiological systems due to its unique transmembrane distribution and broad-spectrum antioxidant capacity [74,75]. The skin, as the body’s largest organ, acts as the primary barrier against environmental stressors, especially ultraviolet (UV) radiation. Photoaging drives wrinkle formation, skin laxity, and hyperpigmentation [76,77,78]. Astaxanthin’s regulatory role in the gut-liver axis has opened new avenues for addressing metabolic disorders and liver diseases [79]. Its ability to cross the blood–brain barrier has sparked considerable interest in its potential for neurodegenerative disease research [80]. Park et al. (2024) reported that *H. pluvialis* extract exhibits potent acetylcholinesterase inhibitory activity [81].

Despite its potent bioactivity, astaxanthin’s poor water solubility and sensitivity to light, heat, and oxygen limit its oral bioaccessibility and shelf-life stability [82]. To tackle these challenges, significant progress has been made in clarifying structure–activity relationships and developing advanced delivery systems [83,84]. Natural astaxanthin from *H. pluvialis* is mainly present in esterified forms, unlike synthetic astaxanthin which is mostly free [85]. Pharmacokinetic studies by Lao et al. (2022) showed that astaxanthin esters have better thermal stability and oral bioaccessibility—this is due to enhanced protection of the chromophore and improved formation of mixed micelles with bile salts during digestion [86]. Notably, cis-isomers (especially 13-cis) accumulate at higher levels in the body than all-trans isomers [87]. Building on this, Villaró et al. (2021) [88] further found that formulations rich in monoesters exhibited stronger antioxidant and anti-inflammatory activities compared to those dominated by diesters or free astaxanthin. This suggests that selective enrichment of monoesters could be beneficial for astaxanthin product development .

Advanced delivery systems directly address the bioaccessibility limitations of astaxanthin, creating a critical link between formulation innovation and clinical application. Composed of solid–liquid lipid matrices, NLC-encapsulated esterified astaxanthin (rich in 13-cis isomers) enhances dermal penetration and bioaccessibility [89]. In UVB-induced skin damage models, NLC formulations outperformed nanoemulsions and nanoliposomes in alleviating photoaging, supporting their potential in clinical dermatological applications. These delivery systems not only overcome astaxanthin’s physicochemical limitations but also optimize the bioactivity of its active forms (monoesters and cis-isomers), directly translating laboratory findings into clinically viable formulations. Traditional oil-based soft capsules have been expanded to liquids, water-dispersible powders, and transdermal delivery systems, meeting diverse clinical application needs.

Peinsipp et al. (2025) provided novel insights into this function: in a D-galactose-induced aging mouse model, administration of *H. pluvialis* powder or extracts significantly altered gut microbiota composition, increasing the abundance of Bacteroidetes and beneficial genera [90]. This elevation in short-chain fatty acid production suppressed hepatic inflammation and oxidative stress, improving liver function indices [91]. Furthermore, Aslanbay Guler et al. (2023) reported that astaxanthin-rich extracts inhibited hepatic stellate cell activation and downregulated α-smooth muscle actin and transforming growth factor-β, effectively slowing the progression of liver fibrosis [92].

In the biomedical and clinical sections, we separate evidence by study tier (in vitro, animal, and human) and standardize reporting by intervention form (whole biomass vs. extract/oleoresin vs. purified astaxanthin; free vs. esterified), dose, duration, formulation, primary outcomes, and safety signals. This structure is intended to prevent conflation of mechanistic findings with clinical efficacy and to clarify which claims are supported by human evidence. Clinically, a randomized, double-blind, placebo-controlled trial by Ha et al. (2024) showed that supplementation with astaxanthin-rich extract significantly improved cognitive performance in healthy middle-aged and elderly individuals, with no observable adverse effects [93]. In oncology research, Mehariya et al. (2020) and Pratap et al. (2022) found that *H. pluvialis* extracts significantly inhibited the proliferation, migration, and invasion of triple-negative breast cancer cells [94,95]. Mechanistically, astaxanthin activated intrinsic apoptotic pathways and downregulated Bcl-2 and mutant p53. Additionally, Liu et al. (2025) demonstrated that astaxanthin enhances splenocyte viability and modulates cytokine secretion in mice, highlighting its potential as an immunomodulatory agent or immune adjuvant [96].

To further boost bioaccessibility, a variety of nanodelivery systems have been developed [97]. In addition, Mirzajani et al. (2025) applied hot-melt extrusion (HME) technology to prepare amorphous solid dispersions of astaxanthin, which significantly improved dissolution rates and anti-inflammatory efficacy in a murine sepsis model [98]. These advances have expanded astaxanthin formulations beyond traditional oil-based soft capsules, enabling the development of liquids, water-dispersible powders, and transdermal delivery systems.

## 5. Existing Controversies and Limitations

Despite the significant progress made in recent years, there are still unresolved scientific and practical challenges in the research and commercialization of *H. pluvialis*-derived astaxanthin [99,100].

High extraction efficiency often comes at the cost of astaxanthin’s molecular integrity—and subcritical water extraction is a typical example of this dilemma. While high yields and strong apparent antioxidant activity were reported at elevated temperatures, detailed analyses later revealed that most native astaxanthin had degraded. The observed antioxidant effects were mainly attributed to Maillard reaction products and heat-stable vitamin E [101]. Similar concerns apply to overly aggressive treatments using ionic liquids or electrotechnologies, which may trigger lipid oxidation and astaxanthin isomerization [102]. Thus, striking an optimal balance between extraction efficiency and bioactivity preservation remains a key technical challenge in the field. A long-standing consensus has favored all-trans astaxanthin as the most bioactive and stable form. However, emerging evidence suggests that cis-isomers—especially 13-cis—are more readily absorbed and accumulate at higher levels in mammalian tissues [103,104]. Some in vitro studies even indicate that cis-rich mixtures exhibit enhanced bioactivity. These findings raise critical questions about current industrial standards that prioritize all-trans purity. At the same time, they highlight the practical challenges posed by the thermodynamic instability and poor shelf-life of cis-isomers, complicating the development of cis-enriched products [105].

While extensive in vitro and animal studies have demonstrated the health benefits of astaxanthin, clinical evidence from human trials remains limited [106,107]. Most randomized controlled trials (RCTs) suffer from small sample sizes and short intervention durations. Robust clinical validation is lacking for therapeutic applications—particularly in oncology and liver fibrosis—and long-term safety data for high-dose astaxanthin supplementation are still insufficient [108,109]. This imbalance between strong laboratory evidence and weak clinical validation continues to hinder astaxanthin’s transition from dietary supplements to more rigorous clinical nutrition or pharmaceutical applications [110].

## 6. Future Directions

In light of the aforementioned challenges, future research and industrial development should prioritize several strategic directions to accelerate the upgrading and sustainable transformation of the *H. pluvialis* industry [111,112]. Current industrial models focus primarily on extracting the approximately 4% astaxanthin from *H. pluvialis* biomass, treating the remaining 96% as waste—a practice that results in significant resource inefficiency. Future development should therefore adopt an integrated biorefinery approach. Growing evidence shows that post-extraction algal residues remain rich in high-quality proteins with balanced amino acid profiles, as well as polysaccharides and dietary fiber [113]. For instance, Zhou et al. (2024) demonstrated that proteins recovered from these residues have excellent emulsifying properties and can serve as natural food emulsifiers, potentially replacing commercial sodium caseinate [114]. Meanwhile, Ambatii et al. (2019) established efficient pathways for converting residual biomass into bioethanol and biomethane [115,116]. Moving forward, next-generation *H. pluvialis* facilities should evolve into multifunctional biorefineries capable of co-producing astaxanthin, functional food ingredients, high-protein feed, and bioenergy. This would distribute upstream cultivation costs across multiple value streams, achieving both economic and environmental sustainability. Future progress in this field is likely to rely less on further increasing astaxanthin yields alone, and more on addressing the fundamental coupling between stress-induced carotenogenesis and cell wall recalcitrance at the cellular level (Table 3).

**Table 3:** Future perspectives and emerging strategies for the sustainable development of *Haematococcus pluvialis* industry.

Future Direction	Key Technology/Strategy	Mechanism and Approach	Expected Outcome/Application	Reference
Energy Co-production	Biofuel Generation	Anaerobic digestion and fermentation of algal waste streams.	Co-production of bioethanol and biomethane; offsets energy costs of cultivation.	[117]
Precision Biomanufacturing	Synthetic Biology (CRISPR/Cas9)	Genetic “redesign”: Downregulating cell wall genes while overexpressing synthesis genes (*BKT/CHY*).	Creation of “thin-walled, high-yield” strains; elimination of mechanical disruption steps.	[118]
Clinical Translation	Evidence-based Medicine	Large-scale, multi-center RCTs targeting aging-related diseases	Validation of therapeutic effects; establishment of standardized analytical methods and quality fingerprinting.	[119]

## Figures and Tables

**Table 1:** Summary of upstream strategies to overcome the “thick wall–high yield” trade-off and improve astaxanthin production in *Haematococcus pluvialis*.

Strategy	Method/Strain	Key Mechanism	Main Outcome/Efficiency	Reference
Metabolic Regulation	exogenous oxaloacetate addition	exogenous OA promoted respiration over photosynthesis.	the metabolite levels in the Embden-Meyerhof-Parnas pathway, pentose phosphate pathway and tricarboxylic acid cycle obviously increased.	[45]
Strain Selection	*H. pluvialis* mutant M5 strain	Maintains motile cell morphology under stress; avoids thick-walled cyst formation.	M5 demonstrated an increase in biomass and astaxanthin productivity by 86.70% and 66.15%.	[46]
High-throughput Screening	A polydimethylsiloxane (PDMS)-based microfluidic device	using the negative phototaxis of the *H. pluvialis* to attain the mutants having high astaxanthin production.	1.17-fold improved growth rate and 1.26-fold increases in astaxanthin production (55.12 ± 4.12 mg g^−1^) in the 100 L photo-bioreactor compared to the wild type.	[47]
Mixotrophy/Cost Reduction	novel fabrication method of alginate hydrogel membrane (AHM)	incorporates cotton gauze into a hydrogel with a low sodium alginate (SA) concentration of 0.5%, utilizing endogenous calcification.	A 70.8% increase in astaxanthin yield	[48]
Biofortification	Sodium acetate (NaAc) supplementation	Provides an exogenous acetate-derived carbon source that directly increases the intracellular acetyl-CoA pool, supporting both fatty acid synthesis (for lipid droplet formation) and astaxanthin esterification; efficacy is often dependent on nitrogen status and culture stage	Enhanced metabolic activity; improved	[49]

**Table 2:** Overview of downstream cell disruption, green extraction technologies, and biorefinery approaches for *Haematococcus pluvialis*.

Method/Technology	Representative system and Conditions	Performance (as Reported)	Key Limitations	Reference
Enzymatic Cell Wall Disruption Technology; Polymer Microcapsule Encapsulation Technology	Enzymatic System; Encapsulation System	Technological Innovation: Pioneering the integration of enzymatic cell wall disruption, extraction, and supercritical encapsulation technologies for astaxanthin stabilization. Process Integrity: Achieving full-chain technological development from algal raw materials to functional products.	Process Complexity: Multi-step processes may increase production costs and operational complexity Scale-Up Challenges: Enzymatic digestion and supercritical encapsulation techniques developed at the laboratory scale may encounter technical barriers during industrial-scale expansion Cost-Effectiveness: The high cost of enzyme preparations and supercritical equipment may impact economic viability	[66]
DES-based aqueous two-phase system (ATPS) pretreatment + subsequent liquid–liquid extraction	35% (*w*/*w*) deep eutectic solvent (choline chloride–urea), 30% (*w*/*w*) dipotassium hydrogen phosphate, 50 °C, pH = 7.5; followed by liquid–liquid extraction at 25 °C	>99% astaxanthin extracted under the above “mild conditions”	solvent reuse/recycling and product-grade compliance need validation at scale	[67]
Recovery of astaxanthin-containing oil by oil partitioning in an oil–acetone–water solution (after nano-dispersion)	Oil Partitioning (Vegetable Oil)	oil-recovery yield 97.8% in 10 g/L solution (partial acetone evaporation + soybean oil addition)	Produces edible astaxanthin-oil directly; reduces cost by ~3-fold (no drying/solvent recovery).	[68]
Mechanochemical Method; One-Pot Room-Temperature Extraction;	Mechanochemical method: Ball milling time 30–60 min, rotation speed 300–500 rpm, aqueous system One-pot method: Room temperature conditions, no additional heating required, processing time 2–4 h	Mechanical-chemical method: Extracts astaxanthin with high purity and no residual organic solvents. One-pot method: Extracts at room temperature with astaxanthin retention rates exceeding 95%.	Cost-effectiveness: New green extraction methods remain more expensive than traditional approaches. Process complexity: While innovative techniques like the one-pot method are highly efficient, they demand stringent process control. Lack of standardization: Different methods lack unified evaluation criteria and process parameters.	[69]

## Data Availability

No new data were created or analyzed in this study. Data sharing is not applicable to this article.
